# Biomechanical analysis of expert anesthesiologists and novice residents performing a simulated central venous access procedure

**DOI:** 10.1371/journal.pone.0250941

**Published:** 2021-04-30

**Authors:** Ignacio Villagrán, Cristóbal Moënne-Loccoz, Victoria Aguilera, Vicente García, José Tomás Reyes, Sebastián Rodríguez, Constanza Miranda, Fernando Altermatt, Eduardo Fuentes-López, Mauricio Delgado, Andrés Neyem

**Affiliations:** 1 Health Sciences Department, Faculty of Medicine, Pontificia Universidad Católica de Chile, Santiago, Chile; 2 Computer Science Department, School of Engineering, Pontificia Universidad Católica de Chile, Santiago, Chile; 3 Department of Biomedical Engineering, Johns Hopkins University, Baltimore, MD, United States of America; 4 Anesthesiology Department, School of Medicine, Pontificia Universidad Católica de Chile, Santiago, Chile; Ohio State University Wexner Medical Center Department of Surgery, UNITED STATES

## Abstract

**Background:**

Central venous access (CVA) is a frequent procedure taught in medical residencies. However, since CVA is a high-risk procedure requiring a detailed teaching and learning process to ensure trainee proficiency, it is necessary to determine objective differences between the expert’s and the novice’s performance to guide novice practitioners during their training process. This study compares experts’ and novices’ biomechanical variables during a simulated CVA performance.

**Methods:**

Seven experts and seven novices were part of this study. The participants’ motion data during a CVA simulation procedure was collected using the Vicon Motion System. The procedure was divided into four stages for analysis, and each hand’s speed, acceleration, and jerk were obtained. Also, the procedural time was analyzed. Descriptive analysis and multilevel linear models with random intercept and interaction were used to analyze group, hand, and stage differences.

**Results:**

There were statistically significant differences between experts and novices regarding time, speed, acceleration, and jerk during a simulated CVA performance. These differences vary significantly by the procedure stage for right-hand acceleration and left-hand jerk.

**Conclusions:**

Experts take less time to perform the CVA procedure, which is reflected in higher speed, acceleration, and jerk values. This difference varies according to the procedure’s stage, depending on the hand and variable studied, demonstrating that these variables could play an essential role in differentiating between experts and novices, and could be used when designing training strategies.

## Introduction

Central Venous Access (CVA) is one of the most common invasive procedures at the Intensive Care Unit [1]. It is prescribed to administer vasoactive drugs, infuse medication, do hemodialysis or hemofiltration and cardiac pacemaking, among other indications [2]. The most common accesses for these procedures are the subclavian vein, internal jugular vein, and femoral vein [1]. There are two main methods to localize the vein that needs to be punctured: anatomical landmarks and ultrasound (US). The first method consists in locating the zone corresponding to the vein that needs to be punctured using some surface landmarks as reference. Then, the zone is penetrated by the guiding needle through the skin. The second method uses real-time ultrasound, relying on 2D images to measure the depth of the vein and surrounding structures to introduce the needle in the targeted zone [2]. Because of its ability to find the vein and calculate the depth of the puncture, the US has shown to be an effective tool for diminishing the risks and complications [3].

Independently from the advantages that a specific technique can bring, the CVA procedure is associated with complications and risks for the patient [4]. These possible complications include: arterial puncture, pneumothorax, hematoma, aneurysm, infections associated with the catheter and risk of developing a thrombosis [1]. The physician’s dexterity is one of the critical factors determining those possible complications during or after the procedure [5]. Therefore, the instructional techniques of teaching and learning CVA focused on reducing the risk of complications to a minimum are essential for the procedure [3].

Amongst the teaching methodologies used, simulation has shown to be a useful strategy to practice procedural skills [6–9]. Simulation has emerged as an effective strategy for CVA, allowing an improvement in students’ technical skills [1, 10], reducing the time in the execution of the procedure [11] and a number of punctures, and increasing the success rate [12]. Additionally, simulation allows students to practice safely, achieving better procedural performance than students who receive traditional training [11, 13].

Any instructional strategy employed requires adequate feedback and assessment, using different types of instruments [14]. Most of them are checklists and global rating scales, relying on a significant subjective component based on observer’s training, attention, or underlying biases. An alternative approach is using objective metrics acquired through technological aids, providing quantitative data about trainee’s performance. A quantitative strategy to determine performance in students is performed through the use of biomechanical analysis [15–17]. This consists in measuring the movement of an individual executing a procedure with markers, cameras, platforms, and other technological tools that allow extracting quantitative data to analyze kinetic and kinematic variables. Some simulated procedures that report biomechanical differences between experts and novices include tracheal intubation and laparoscopy [16, 17]. The analyzed variables include speed, acceleration, and jerk to objectively evaluate procedural skills of experts and novices [17], and hand movement as a reference to determine the performance and competence of an operator performing a simulated procedure [11, 12].

Clinical simulation has proven to be an effective technology for CVA skills acquisition [1, 10]. However, since CVA is a high-risk procedure that requires a detailed teaching and learning process to ensure trainee proficiency, it is necessary to determine objective differences between the experts’ and the novices’ performances that can guide novice practitioners during their training process and teachers in building appropriate simulation strategies. The aim of this study is to advance towards the objective measurement and characterization of variables that could be relevant in the simulated training of the CVA procedure. The primary goal is to compare the biomechanical variables of experts and novices during the performance of a simulated CVA.

## Material and methods

### Participants

To determine the sample size, we considered previous study by Clinkard et al. [15], in which experts and novices were compared on a movement variable (path length of the right hand). A cohen d = 1.89 was obtained for the effect size of the difference between groups. Based on this, a power of 80%, an alpha of 0.05, a two-tailed test, to perform a comparison of independent groups using the Mann-Whitney test, it was necessary to recruit 12 people (6 in each group). An additional 15% were selected for possible loss, so it was decided to recruit 14 people, 7 in each group. Although this is a pilot study, this sample size is similar to other studies comparing experts with novices with similar objectives [15, 17–19].

The study protocol was reviewed and approved by the Institutional Review Board (IRB) at Pontificia Universidad Católica de Chile (PUC). Two groups of participants were recruited, seven specialist physicians, who were experts in the CVA technique, and seven first-year residents in Internal Medicine and Anesthesiology specialties. All participants gave their written informed consent. For the image of a participant shown in the study, the individual has given written informed consent (as outlined in PLOS consent form) to publish these case details.

Each subject was contacted via email. Once they accepted to participate, they were sent general instructions about the simulated CVA technique, a video demonstration of the procedure, and two CVA papers for further reading. At the beginning of the session, each participant was interviewed to obtain their personal data and experience with the procedure.

### Procedure

#### 3D motion capture

**Fig 1:**
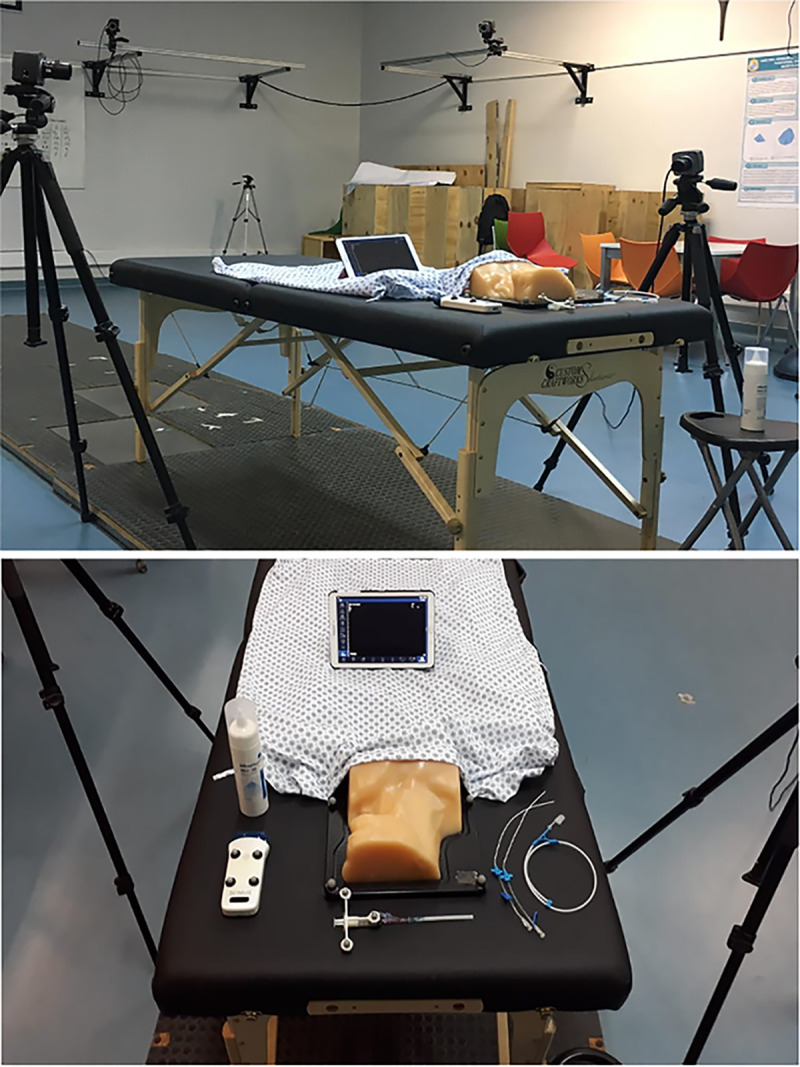
The set-up of simulated scenario in Motion analysis laboratory.

**Fig 2:**
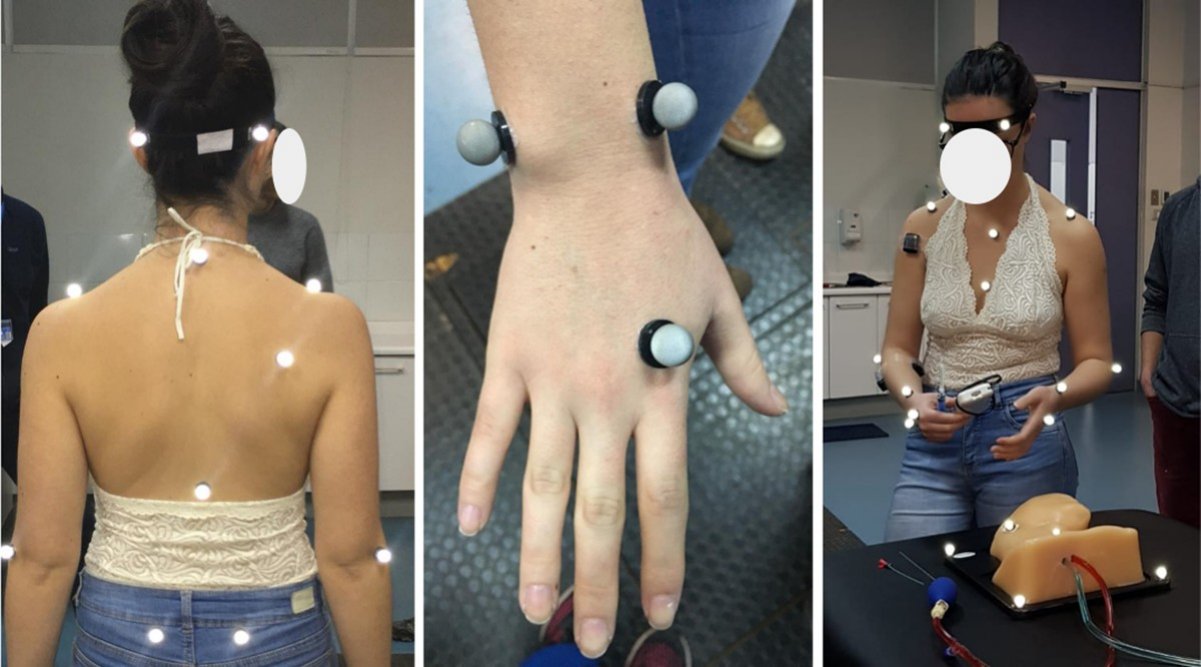
Placement of markers on the subject.

The participants’ biomechanics during a CVA simulation procedure were obtained in the Motion Laboratory Analysis at PUC. A simulated CVA scenario was set up in the laboratory for this purpose. A stretcher was installed, on which there were the phantom covered by a sheet, the syringe, the trocar, 2 catheters of different sizes, the guide, the US transducer, and the gel (Fig 1). Motion data were collected by eight motion capture cameras and two optical cameras synchronized at 100 Hz sampling frequency, using the Vicon system and Vicon Nexus software (Vicon Motion System). Throughout the session, 19 reflective markers were positioned on the surface of the participant’s body, according to the Vicon Plug-In-Gait model [20]. Only the upper limb part of this model was considered, including markers on the trunk and upper extremities (Fig 2). Once the markers were installed, the participants performed the simulated procedure on an Internal Jugular Central Line Ultrasound Manikin (Blue Phantom®, Redmond, WA). This simulation model allows the operator to differentiate between arterial puncture and venous puncture since the simulated arterial blood is red and the simulated venous blood is blue.

The biomechanical variables obtained to evaluate the participants’ ability were speed (mm/s, the first derivative of the position), acceleration (mm/s2, the second derivative of the position), and jerk (mm/s3, the third derivative of the position) of each hand. Since these variables depend on the direction, for comparison, they were transformed to scalar values by applying the average absolute value during each performance. Also, the procedural time (s) was analyzed.

#### Motion analysis

**Fig 3:**
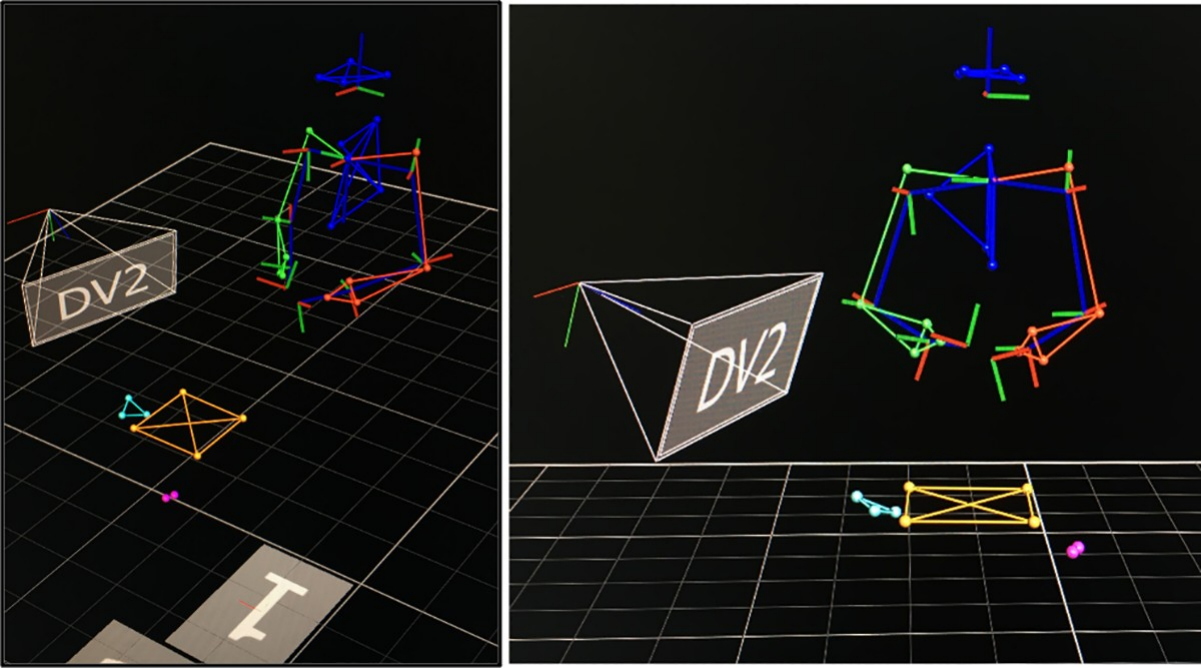
Marker reconstruction in 3D motion capture software.

After the measurements, a 3D reconstruction of the motion data captured by the markers was performed (Fig 3), and then the data was exported for further analysis. For analysis, the procedure was divided into four stages defined according to key moments and milestones during the conduct of the CVA, based on previous studies [21]. Stage 1 began when the ultrasound transducer contacted the phantom, performed the scanning of the anatomic area, and ended when the participants took the syringe. Stage 2 began with the syringe’s contact with the phantom and ended when the syringe was withdrawn from the mannequin. Stage 3 began when the participants began to pass the wire guide and ended when they took the catheter. Finally, Stage 4 began when the participants threaded the catheter and ended when the participants left the syringe on the table.

### Statistical analysis

First, a descriptive analysis of the data was performed, normality was evaluated with the Shapiro-Wilk test and the results were plotted. Subsequently, a multilevel linear model with random intercept was used to analyze differences by group (novice and expert), hand, and stage (first to fourth). The estimation of the model variance was done through a restricted maximum likelihood (REML) because the same parameter (velocity, acceleration, and jerk) measurements would be correlated by analyzing both hands simultaneously.

In the case of the models, according to each hand, these were of linear type, adjusted by the existing correlation between stages for the same subject, estimating cluster-robust standard errors. The models were built progressively, from univariable, and multivariable with interaction. The interaction model allowed to evaluate if there were differences in the procedure session according to the group (novice and expert). This is valid for analyzing differences between groups, segments, and their interaction for acceleration, speed, and jerk.

## Results

### Participants

A total of 14 participants completed the study, 7 experts and 7 novices. The experts’ average age was 39 years (SD = 4.3) and of the novices was 28.6 years (SD = 3.03). In the experts’ group, 71.4% were men and 28.6% women whereas among the novices, 85,7% were men and 14.3% women. Furthermore, 57% of novices were in the anesthesiology program, 29% in the emergency program, and 14% in the internal medicine program. Additionally, the novice group had performed less than five CVA procedures on real patients, while the expert group had performed an average of more than 300 procedures on real patients. All participants declared their right hand as their dominant hand.

### Quantitative analyses

#### Time

**Fig 4:**
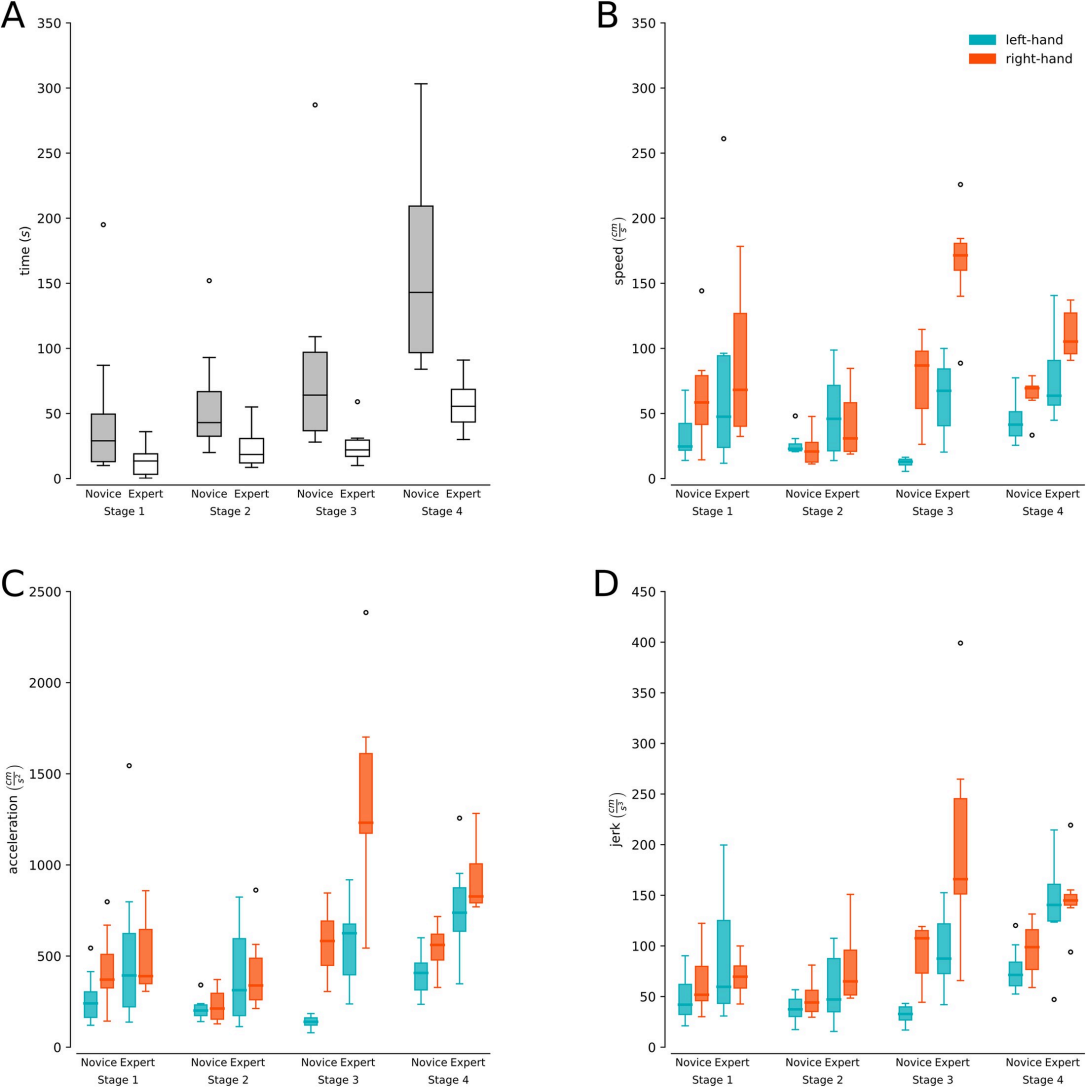
Procedural time and biomechanical variables. **(A)** Duration (y-axis) for each of the stages of the CVA simulation for experts (gray boxes) and novices (white boxes). **(B** and **C)** Speed, acceleration, and jerk (y-axis) for each of the CVA simulation stages for experts and novices separated by hand (light blue boxes left hand and orange boxes right hand).

The expert group took less time to carry out the procedure for Stage 1 (Experts M = 13.2, SD = 12.8 vs Novices M = 56.9, SD = 66.0), Stage 2 (Experts M = 23.1, SD = 16.4 vs Novices M = 63.2, SD = 45.0), Stage 3 (Experts M = 25.1, SD = 16.6 vs Novices M = 94.6, SD = 89.5) and Stage 4 (Experts M = 58.0, SD = 20.4 vs Novices M = 161.92, SD = 74.0). The overall time distribution for each stage/group is presented in Fig 4A. Table 1 describes the univariable and multivariable linearity analysis for stage time to determine the time differences according to the group, stage, and their interaction. The third stage was chosen as a reference in the statistical models because a greater significant difference was observed in relation to the other stages. The multivariable model resulted in significant differences between experts and novices (p<0.05). On the other hand, the group-stage interaction was not significant for time, demonstrating that the differences between experts and novices in time do not vary according to the stage. Finally, it is observed that the model’s predictive capacity by adding the interaction was 46%.

**Table 1:** Univariable and multivariable linear analysis to determine differences in time by group and its interaction with stage.

	*Univariable models*		*Multivariable model with interaction*	
Variable	Coefficient	*p-value*	Coefficient	*p-value*
Group				
Novice	Reference	-	Reference	-
Expert	**-59.38 (-80.94- -37.83)**	**p<0.001**	**-42.54 (-77.01–8.06)**	**p<0.05**
Group-Stage interaction^a^	-	-		0.129

^a^The value of statistical significance is obtained by applying a Wald test.

There was a significant interaction between the hand and the procedure stage (X2(3) = 11.15; p<0.05), therefore it was decided to stratify the following models according to each hand. The analysis results in differences in the behavior in each session according to the hand evaluated.

#### Speed

Table 2 shows that the multivariable analysis resulted in significant differences between experts and novices for the left (p<0.01) and right (p<0.05) hand, and Fig 4B presents the speed distributions for stage/group separated by left and right hand. Also, we can observe that the group-stage interaction for speed was not significant for both the left (p = 0.522) and right (p = 0.099) hand, demonstrating that the differences between experts and novices in speed do not change significantly according to the stage. Finally, it is observed that the model’s predictive capacity by adding the interaction was 30% for the left hand and 34% for the right hand.

**Table 2:** Univariate and multivariate linear analysis to determine differences in speed by group, and its interaction with stage, for both hands.

***Left hand***				
	*Univariate models*		*Multivariate model with interaction*	
Variable	Coefficient	*p-value*	Coefficient	*p-value*
Group				
Novice	Reference	-	Reference	-
Expert	38.20 (13.68–62.72)	p<0.01	44.29(20.80–67.79)	p<0.01
Group-Stage interaction	-	-		p = 0.522
**Right hand**				
	*Univariate models*		*Multivariate model with interaction*	
Group				
Novice	Reference	-	Reference	-
Expert	58.89 (12.20–105.58)	p<0.05	764.39(257.87–1270.90)	p<0.01
Group-Stage interaction^a^	-	-		p = 0.099

^a^The value of statistical significance is obtained by applying a Wald test.

#### Acceleration

Table 3 shows significant differences in the multivariate analysis between experts and novices for the left (p<0.01) and right (p<0.01) hand, and Fig 4C presents the acceleration distributions for stage/group separated by left and right hand. Additionally, for the left hand, the group-stage interaction was not significant for acceleration (p = 0.417). On the contrary, a statistical interaction between group and stage was shown for the right hand (p<0,05), demonstrating that its differences change significantly according to the stage only for the right hand. Finally, it is observed that the model’s predictive capacity by adding the interaction was 43.3% for the left hand and 35% for the right hand.

**Table 3:** Univariate and multivariate linear models to determine differences in acceleration by group, session, and their interaction, for both hands.

***Left hand***				
	*Univariate models*		*Multivariate model with interaction*	
Variable	Coefficient	*p-value*	Coefficient	*p-value*
Group				
Novice	Reference	-	Reference	-
Expert	314.47 (141.64–487.30)	p<0.01	382.28 (226.06–538.50)	p<0.001
Group-Stage interaction	-	-	-	0.417
***Right hand***				
	*Univariate models*		*Multivariate model with interaction*	
Group				
Novice	Reference	-	Reference	-
Expert	458.64 (63.05–854.24)	p<0.05	764.39 (257.87–1270.90)	p<0.01
Group-Stage interaction^a^	-	-	-	p<0.05

^a^The value of statistical significance is obtained by applying a Wald test.

#### Jerk

When looking at the multivariate analysis (Table 4), significant differences were found between experts and novices in both the left (p<0.01) and right (p<0.05) hand, and Fig 4D presents the jerk distributions for stage/group separated by left and righthand. For jerk, the group-stage interaction was significant for the left hand (p<0.05) but not significant for the right hand (p = 0.068), demonstrating that the differences in jerk change significantly according to the stage only for the left hand. Finally, it is observed that the model’s predictive capacity by adding the interaction was 50.2% for the left hand and 30% for the right hand.

**Table 4:** Univariate and multivariate linear models to determine differences in jerk by group, session, and their interaction, for both hands.

***Left hand***						
	*Univariate models*			*Multivariate model with interaction*		
Variable	Coefficient		*p-value*	Coefficient		*p-value*
Group						
Novice	Reference		-	Reference		-
Expert	47.27 (16.85–77.69)		p<0.01	55.51 (28.73–82.29)		p<0.01
Group-Stage interaction	-		-	-		p<0.05
**Right hand**						
		*Univariate models*			*Multivariate model with interaction*	
Group						
Novice	Reference		-	Reference		-
Expert	63.36 (-0.83–127.56)		0.053	101.72 (8.14–195.32)		p<0.05
Group-Stage interaction^a^	-		-	-		0.068

^a^The value of statistical significance is obtained by applying a Wald test.

## Discussion

The biomechanical analysis results support the hypothesis that there are statistically significant differences between experts and novices regarding time, speed, acceleration, and jerk during a simulated CVA performance. These differences vary significantly by the procedure stage for right-hand (dominant) acceleration and left-hand (non-dominant) jerk, demonstrating that stage characteristics could play an essential role in differentiating between experts and novices.

The differences between experts and novices found in our study coincide with those reported in CVA with other motion analysis systems for instruments and/or body parts, such as hand-track motion devices [15, 22], gaze pattern analysis [23], and video analysis for eye-hand coordination [24]. Specifically for 3D body motion tracking systems, as in our study, differences have been reported using body assessment in tracheostomy [17]. To our knowledge, this is the first study that analyzes hand biomechanical differences between experts and novices using a 3D capture system for the central venous access procedure.

Considering the overall procedure, the experts took less time to complete the procedure and its stages, which coincides with the differences found between experts and novices regarding the procedural time [10, 15, 17, 18]. Furthermore, although it is expected that novices become faster as they receive training [25], we believe that the improvement in this variable is not sufficient to provide feedback to novices on the relevant aspects to become CVA experts, making it necessary to determine the performance of other objective indicators such as biomechanical variables. Moreover, shorter procedural times are a consequence of higher expertise, and taken alone, they do not predict performance in itself.

Regarding biomechanical variables, the experts presented higher values of speed, acceleration, and jerk. By contrast, in tracheostomy, it was shown that experts demonstrated lower speed, acceleration, and jerk [17]. Studies in other procedures have shown results similar to ours but analyzing the instrumental movement of the procedure and not the hands, finding for experts higher values in speed, acceleration, and jerk in the case of laparoscopy [19, 26], and higher values in velocity and acceleration, and lower values in jerk in the case of transcatheter aortic valve implantation [18].

We believe that these mixed results are mainly due to two factors: the nature of the procedure and the expertise. On the one hand, the CVA’s specific characteristics promote rapid and repeated motor gestures, especially the guidewire’s manipulation (stage 3), where the speed of execution is essential to perform the procedure in adequate time. On the other hand, the expertise is usually related to jerk, which is associated with the movement path’s smoothness and motor control: the more pronounced the jerk, the less smooth the trajectory [17]. It is tempting to associate expertise with lower jerk values; however, motor planning between experts and novices is not necessarily the same. For example, lower jerk values in novices have been associated with the cautious execution of their movements due to their inexperience [19].

Additionally, we also found differences between experts and novices according to the stage of the procedure and hand, specifically for right-hand acceleration and left-hand jerk. The literature has already highlighted the importance of training bimanuality, emphasizing that the use of both hands is greater in experts than in novices and that it is important for optimal surgical procedures [27]. Our results also support this claim. For example, in the case of acceleration, stage 3 stands out since the novices presented speed and acceleration values close to zero in contrast to the experts that presented accelerations in their left hand comparable to the right hand of the novices. Also, the experts’ acceleration in the right hand was more than twice as high as that of a novice, demonstrating the experts’ confidence in performing the CVA procedure and the use of bimanual strategies in contrast to novices, who tended to use only their right hand. Procedurally, this could translate into greater efficiency when manipulating the guide-wire, one of the stages of the procedure where novices make the most mistakes [21]. Finally, the significant difference in the jerk at the left-hand (non-dominant) level is a strong indicator of the experts’ bimanuality. The experts’ left-hand jerk values were higher than novices’ mainly due to the little use of the non-dominant hand (quantified mainly through jerk amount) among the novices during the procedure. This effect is more noticeable in stages 2 (syringe puncture) and 3, given the low speed (close to zero) that novices presented in these stages. Meanwhile, stage 4 (catheter placement and guide-wire removal) presented similar biomechanical values between hands, but with notable differences between groups. This stage highlights that expertise allowed the experts to accomplish specific tasks more efficiently and more quickly [25].

Furthermore, it is worth noting that CVA also requires adequate handling of instruments (ultrasound transducer, syringe, guide-wire, and catheter) and visual coordination with an external screen in the case of US visualization. The latter occurs mainly in stage 1 of the procedure (scanning the phantom with the US), in which our results show no differences. Possibly because the stage’s discriminant features are not on the hands themselves but on the instrument movement and the visual coordination. Indeed, motion analysis of procedures that depend heavily on instrument manipulation focuses on the instrument movement’s biomechanics instead of the hand’s motion [19, 26]. Also, when visual coordination is a critical component of the procedure, other variables are measured, such as gaze patterns [23]. We believe that both analyses (instrument motion and eye-tracking) are necessary to further describe differences between experts and novices in stage 1. It is worth mentioning that given the small sample size, no multiple contrast analysis was performed to determine specific statistical differences by stage, which leaves an exciting opportunity for future work.

## Conclusion

Biomechanical analysis using a 3D motion capture system allowed the detection of objective and significant differences between experts and novices during the CVA procedure. The results have demonstrated that experts take less time to perform the CVA procedure, which was reflected in higher speed, acceleration, and jerk values. This difference varies according to the procedure’s stage depending on the hand and variable studied. This study may serve as a starting point for studies that wish to deepen this analysis with more subjects and for teachers seeking to optimize their teaching-learning process of procedural skills in CVA.
